# Electrochemical Preparation of a Molecularly Imprinted Polypyrrole-modified Pencil Graphite Electrode for Determination of Ascorbic Acid

**DOI:** 10.3390/s8095792

**Published:** 2008-09-18

**Authors:** Levent Özcan, Mutlu Şahin, Yücel Şahin

**Affiliations:** Anadolu University, Faculty of Science, Department of Chemistry, 26470 Eskişehir, Turkey; E-mails: leventozcan@anadolu.edu.tr (L.Ö); mutlus@anadolu.edu.tr (M.Ş.).

**Keywords:** Ascorbic acid, polypyrrole, pencil graphite electrode, molecularly imprinted polymer

## Abstract

A molecularly imprinted polymer (MIP) polypyrrole (PPy)-based film was fabricated for the determination of ascorbic acid. The film was prepared by incorporation of a template molecule (ascorbic acid) during the electropolymerization of pyrrole onto a pencil graphite electrode (PGE) in aqueous solution using a cyclic voltammetry method. The performance of the imprinted and non-imprinted (NIP) films was evaluated by differential pulse voltammetry (DPV). The effect of pH, monomer and template concentrations, electropolymerization cycles and interferents on the performance of the MIP electrode was investigated and optimized. The molecularly imprinted film exhibited a high selectivity and sensitivity toward ascorbic acid. The DPV peak current showed a linear dependence on the ascorbic acid concentration and a linear calibration curve was obtained in the range of 0.25 to 7.0 mM of ascorbic acid with a correlation coefficient of 0.9946. The detection limit (3σ) was determined as 7.4×10−5 M (S/N=3). The molecularly-imprinted polypyrrole-modified pencil graphite electrode showed a stable and reproducible response, without any influence of interferents commonly existing in pharmaceutical samples. The proposed method is simple and quick. The PPy electrodes have a low response time, good mechanical stability and are disposable simple to construct.

## 1. Introduction

Ascorbic acid (AA, vitamin C) is an important water-soluble substance present in many biological fluids, fruit juices, pharmaceuticals, soft drinks, vegetables, etc. AA is also added to foodstuffs as an antioxidant for stabilization of color and aroma, as well as for prolonging the life of commercial products [1]. AA can also be used as free-radical scavenger, important in the treatment of cancer and Parkinson's disease. The accurate determination of AA concentrations is thus essential for food quality and health care. Fort this reason many analytical procedures, for example, indirect spectrometry, solid-phase iodine method and liquid chromatography, have been proposed for AA detection at many different concentration levels in food, drugs and plants [2-5].

AA is electroactive and can be oxidized at positive potentials. However the kinetics of the oxidation are “sluggish” and the oxidation products tend to lead to electrode fouling [6]. Therefore, modified electrode systems have been investigated for the selective determination of AA both at acidic and neutral pHs [7]. The electrooxidation of AA has been investigated on various modified electrodes, such as polypyrrole [6, 8-12], polyaniline [13-16], etc. and the resulting electrodes have been used for the determination of AA, but it is often difficult to compare results obtained in separate studies to the scarce information concerning some characteristics of the conducting polymer layers such as redox charge (or thickness) of the polymer coating [17]. A review on electrochemical analysis of AA using conducting polymer modified electrodes has been published [18].

Conducting polymers can be used as solid-contact electrodes. This type of sensors, by eliminating the internal filling solution, provide new advantages, for instance, good mechanical stability, simplicity and possibility of miniaturization. Polypyrrole has received the most attention owing to its convenience of preparation, high stability and wide range of applications. PPy shows its electric conductivity and electrochemical redox activity even in pH-neutral solutions, which allows the entrapment of a wide range of biocatalysts [19]. They have been used as detectors in hydrodynamic separation systems such as chromatographic methods. Wang *et al.* demonstrated that nucleic acids can be detected at polypyrrole (PPy)-modified flow detectors [20], and attributed the response to capacitance changes accrued from adsorption–desorption on the PPy surface. Our group reported the use of polypyrrole and overoxidized polypyrrole as a potentiometric detector for determination of inorganic anions and cations by ion chromatography [21]. Jiang and Wang employed conducting polymer (PPy) molecular interfaces for detecting biotechnologically important synthetic oligonucleotides (ODNs) in the presence of chromosomal DNA [22].

Molecular imprinting techniques are becoming more commonly accepted as useful methods for the recognition and isolation of key biological target molecules. This attention can be explained by the serious potential advantages of using molecularly imprinted polymers (MIPs) in place of natural receptors and enzymes such as their superior stability, low cost and easy preparation. The general principal of molecular imprinting is based on such a process where monomers are polymerized in the presence of a target analyte (the imprint molecule) which acts as a molecular template. This procedure can be accomplished via either reversible covalent bonding or non-covalent interactions between monomers and imprinting molecules. The construction and processing of MIPs for extraction, chromatography and sensor applications has typically involved casting, crushing, sieving and electropolymerization of these materials. The electropolymerization methods provide a simple and rapid technique of controlling the thickness of the conductive polymer film grown adherent to a transducer of any size and shape. Various types of electrosynthesized polymers based on molecular imprinting have been reported in the literature, including polypyrrole [23] and a copolymer of aniline with *o*-phenylenediamine [24].

We chose pencil graphite as the material of the electrode because the pencil graphite electrode (PGE) has a larger active electrode surface area and is therefore able to detect low concentrations and/or volumes of the analyte. This is significant when only small amounts of analyte are available. Moreover, disposable PGEs have been used by virtue of their high electrochemical reactivity, good mechanical rigidity, low cost, low technology, low background current, wide potential window, chemical inertness and ease of modification, renewal, and miniaturization [25]. The PGE, when combined with a more highly sensitive and accurate voltammetric technique such as differential pulse voltammetry, becomes an attractive electrode for trace analysis. We reported the electrochemical oxidation of ds-DNA [26] and preparation of a molecularly imprinted polymer film for paracetamol on the surface of the nanofiber polypyyrole modified PGE [27]. Disposable PGEs were employed in the development of enzymeless biosensors for glucose based on overoxidized polypyrrole nanofiber electrode modified with cobalt (II) phthalocyanine tetrasulfonate by our group [28]. This modified electrode (PG/OPPyNF/CoPcTS) shows excellent stability and can be used as an electrode directly for the determination of glucose in NaOH solution without enzyme loading.

The use of a differential pulse voltammetry to determine the AA using pencil graphite electrode prepared by imprinting electropolymerization was reported for the first time in this work. AA was chosen as template molecule because of its popularity and electroactivity. Its successful application to the determination of AA in commercial pharmaceutical samples has been demonstrated.

## 2. Results and Discussion

The properties of the MIP and NIP polypyrrole electrodes depend on the pH of the solution, electropolymerization cycles and the composition of the polymer, i.e., concentration of monomer and template.

### 2.1. Electropolymerization of molecularly imprinted polypyrrole

**Figure 1.**
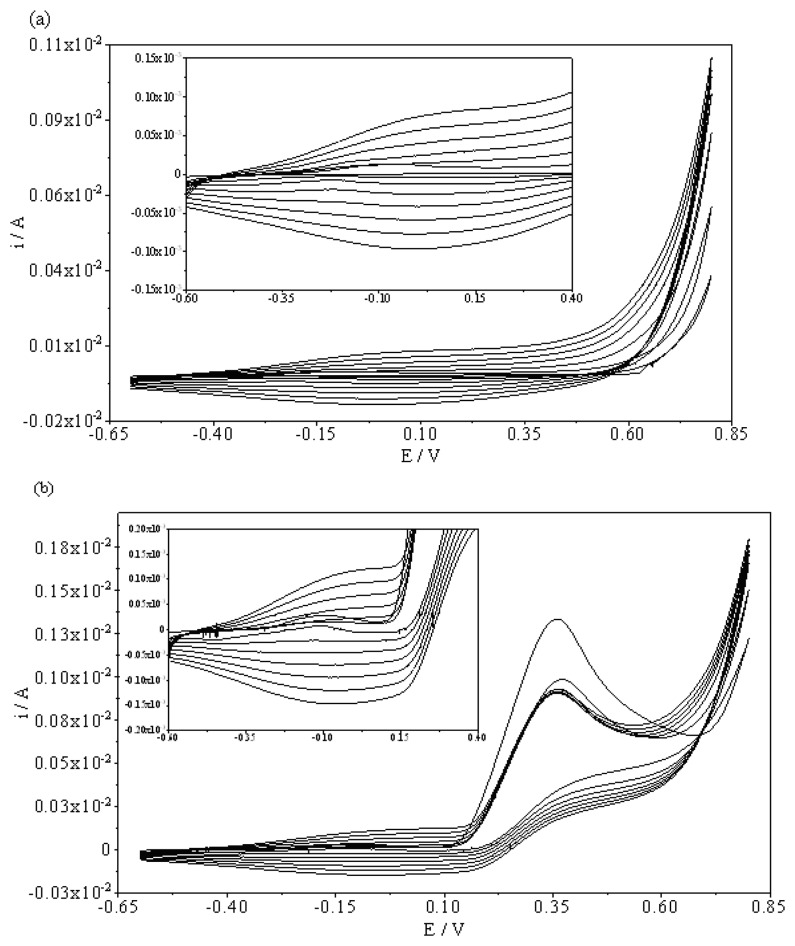
Cyclic voltammograms taken during the electropolymerization of pyrrole (0.025 M) in the absence (a) and presence of 10 mM AA (b) onto a PGE. Scan rate: 100 mV/s. Supporting electrolyte: 0.1 M LiClO_4_. Number of scans: 7.

Electrooxidation of the pyrrole monomer occurs at the anode and the resulting polymer deposits onto the surface of the PGE. Figure 1a shows the cyclic voltammograms taken during the electropolymerization of pyrrole (0.025 M) onto a PGE. The formation and growth of the polymer film can be easily seen in this figure. The peaks due to the oxidation and reduction of the film increase in intensity as the film grows. A broad oxidation peak was observed at the peak potential of +0.15 V and reverse cathodic peak was seen at a peak potential of +0.00 V. During the electropolymerization process, AA template molecules diffuse towards the surface of the PGE and trapped in the polymer matrix as a result of the ability of these molecules to interact with the pyrrole units (Figure 1b). Because the polymerization solution was not stirred, the mass transfer occured by a diffusion controlled process. The creation of the molecular imprints was favored by the diffusion of the electroactive template, generating a far higher number of recognition sites than a non-electroactive template. Figure 1b shows the cyclic voltammograms taken during the electropolymerization of pyrrole (0.025 M) in the presence of AA (10 mM). The oxidation peak potential of polypyrrole shifted to more cathodic potentials, from 0.15 V to 0.10 V, in the presence of AA. A new oxidation peak at +0.35 V indicates that the template is becoming part of the polymeric chain [29].

**Figure 2.**
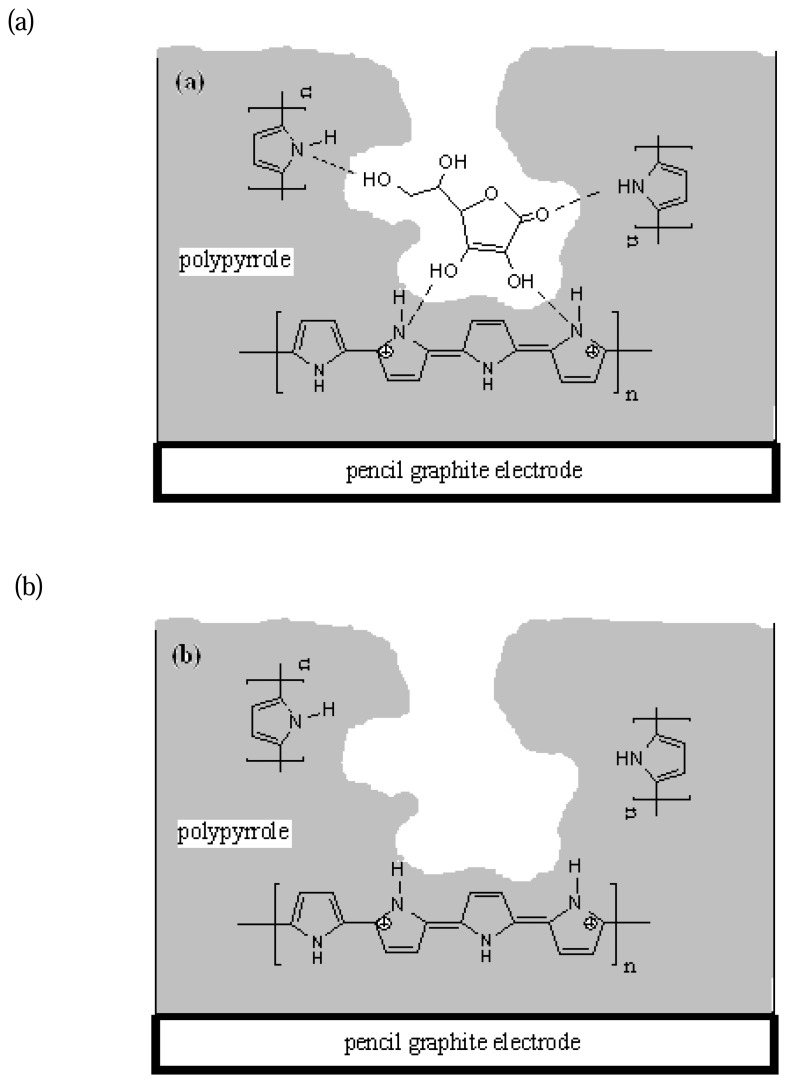
Schematic representation of (a) imprinting and (b) removal of AA from pAA imprinted polypyrrole modified pencil graphite electrode.

From a theoretical point of view, at least two hydrogen bond formations are possible: (1) between the oxygen atom in the C=O group of the AA molecule and the hydrogen atom in the N-H group of the pyrrole units, and (2) the hydrogen in the hydroxyl group of AA molecule and the nitrogen atom of the N-H group of pyrrole units. A schematic representation of imprinting and removal of AA from an AA imprinted polypyrrole modified pencil graphite electrode is shown in Figure 2. The template was removed by immersing the MIP film-coated electrode into 0.05 M phosphate buffer solution with agitation provided by a magnetic stirrer. If longer times were given for extraction an increased number of the imprinted cavities may have been destroyed giving rise to the poorer selectivity [30]. The ideal extraction time was found to be approximately 15 min. This imprinting process creates a microenvironment for the recognition of AA molecule based on shape selection and positioning of the functional groups.

### 2.2. Effect of pH

**Figure 3.**
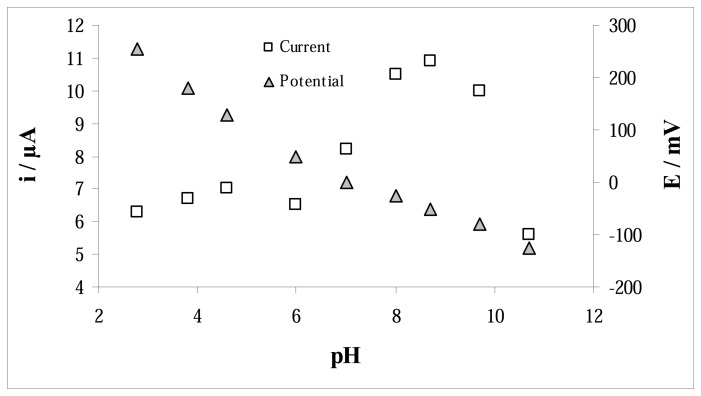
Effect of the pH on the MIP modified PGE response.

The pH of the medium has a significant influence on the peak current and peak potential of the electrochemical oxidation of AA on the polymeric film. Figure 3 shows the dependence of the DPV peak current and potential of AA on the pH, in the range of 3-11. As can be seen, the AA signal shifted to more cathodic potentials as the pH increases. The peak current for AA oxidation increased and shifted to more positive potentials with increase in pH. The highest current value was obtained at pH value of 8.5, with a 0.05 M phosphate and 0.1 M KCl, giving an oxidation peak at −40 mV. No further improvements were observed at higher ionic strength.

### 2.3. Effect of AA concentration on PGE response

**Figure 4.**
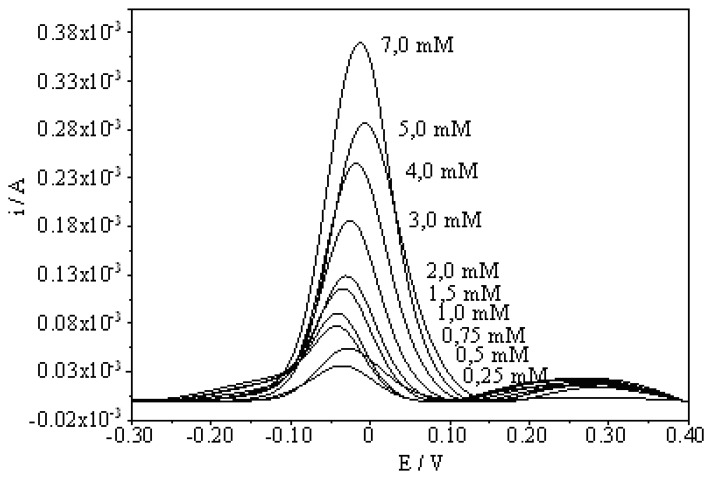
Differential pulse voltammograms of varying ascorbic acid concentrations in the range 0.25 to 7.0 mM at pH 8.5.

A series of DPVs was recorded at various concentrations of ascorbic acid at pH 8.5 at the surface of MIP electrode (Figure 4). The response of the MIP electrode to ascorbic acid was found to increase with increasing ascorbic acid concentration.

**Figure 5.**
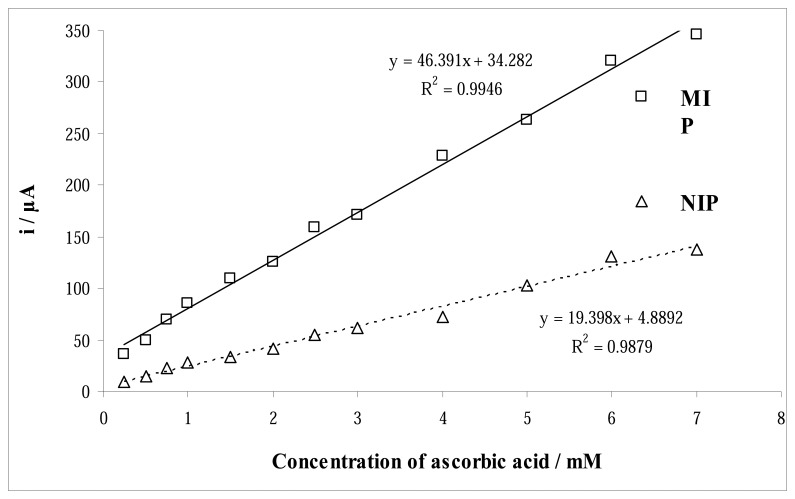
MIP and NIP electrode response to the concentration of ascorbic acid.

The DPV of increasing concentration of ascorbic acid at pH 8.5 at the surface of MIP and NIP electrodes was studied. The intensity of the oxidation peak current of AA depends on its concentration (Figure 5). The linear relationship was observed between peak current versus concentration of AA, in the concentration range of 0.25-7.0 mM, with a correlation coefficient of 0.9946 and 0.9879 for MIP and NIP electrodes, respectively. The detection limit (3*σ*), taken as the concentration that gave a signal equal three times of standard deviation of blank signal calculated from the analytical graph, was 7.4×10−^5^ M.

### 2.4. Effect of the monomer concentration

To determine the effect of monomer concentration on the response of both MIP and NIP to AA, the films were grown in solutions of constant concentration of AA and varying pyrrole concentrations in the range of 25-500 mM by cycling potential between −0.60 V and +0.80 V.

**Figure 6.**
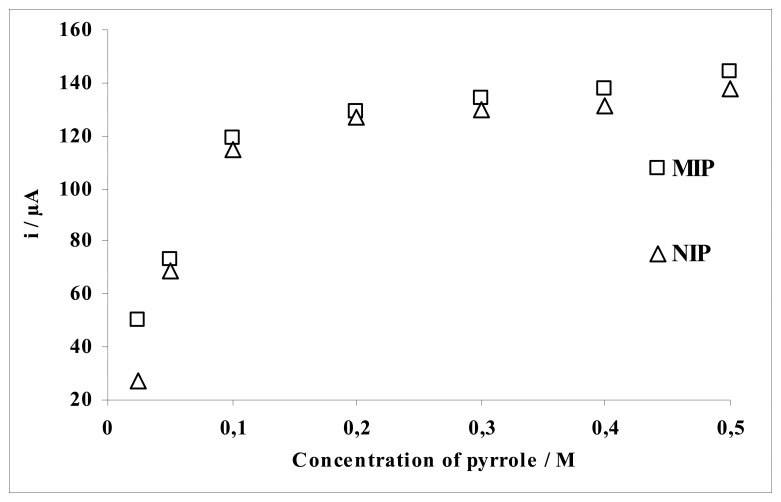
Effect of the monomer concentration.

Figure 6 shows the variation of the monomer concentration as a function of the current values for AA. The monomer concentration should be proportional to the thickness of the deposit and amount of imprinted molecule (template) in the polymeric matrix. The current difference between the MIP and NIP electrode for AA should be as high as possible. In addition, the signal of the NIP electrode should be nearby zero. It can be concluded that the optimum monomer concentration under these conditions was about 25 mM.

### 2.5. Effect of the electropolymerization cycles

**Figure 7.**
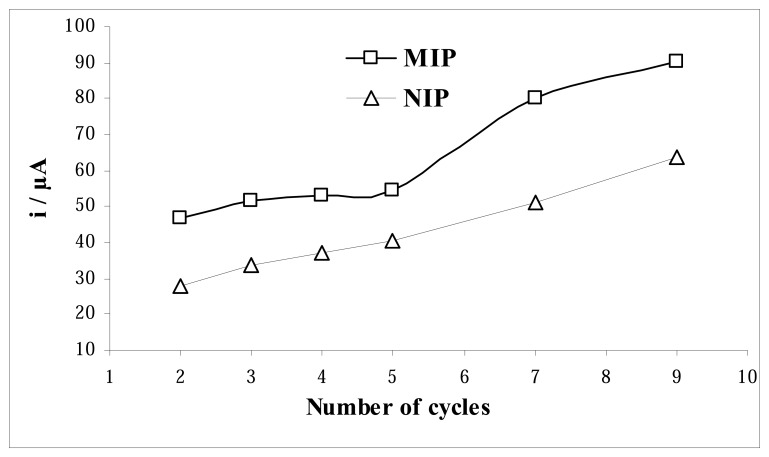
Effect of the electropolymerization cycles.

The optimum number of cycles applied to the cell, for both MIP and NIP, during the electropolymerization was found to affect the sensitivity and linearity of the sensor. In order to obtain a sensing layer of the electrode, a series of experiments were performed by the electrodes fabricated with different numbers of cycles. The template would not be adequately embedded for the resulting surface, after removing of AA, to have well-defined recognition structures with too few cycles. On the other hand, higher cycles lead to more extensive electropolymerization, and therefore to the formation of thicker sensing film with less accesible imprinted sites. The response of the MIP and NIP electrodes to AA was found to increase with increasing the number of cycles (Figure 7). The highest current difference between the MIP and NIP electrode for AA was obtained by applying 7 cycles in the electropolymerization. Therefore the optimum polymerization cycles was found to be 7.

### 2.6. Effect of the template concentration

**Figure 8.**
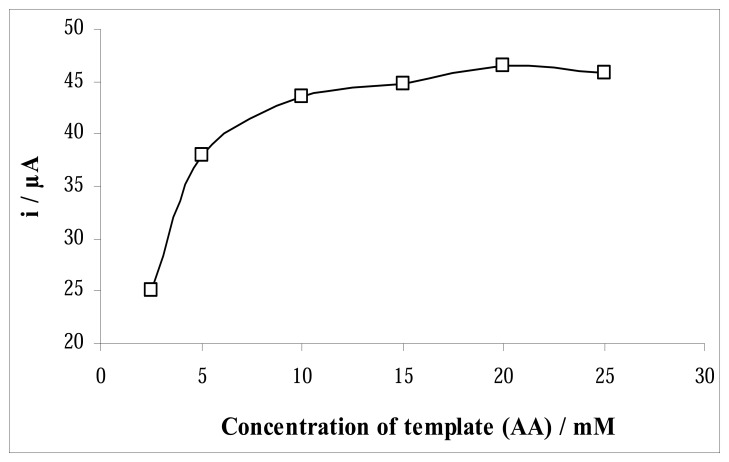
Effect of the template concentration to MIP response.

If the AA template is successfully incorporated into the surface of the polymer matrix during its formation and subsequently extracted, it should leave an imprinted polymeric surface architecture complimentary to itself formed in the polymer film. The effect of the template concentration during the film electrodeposition was shown in Figure 8. The response of the MIP electrode to AA increases with the increase of the template concentration between 2.5 and 10 mM. When the template concentration was higher than 10 mM, the electro-oxidation current almost has no change. At this stage the amount of ascorbic acid arrived at the MIP electrode does not change. Based on the results, the optimum template concentration of was chosen as 10 mM.

### 2.7. Effect of interferents

The electrooxidation of AA in the presence of some possible interfering substances, like glucose, dopamine and uric acid, under similar conditions was also studied at pH 8.5 on the surface of MIP electrode. These substances are present in biological fluids and may interfere with the determination of AA through conventional methods. Dependence of the oxidation current of each interferent in different concentration (0.5-5.0 mM) at a fixed concentration of ascorbic acid (0.5 mM) was investigated using DPV. The results are given in Table 1. At high concentrations of these interferents the variation was within +5.56/−10.4 µA relative to that in their absence. That is, the electropolymerized-molecularly imprinted polypyrrole modified pencil graphite electrode can recognize the AA molecules by means of shape selection and the size of functional groups.

### 2.8. Analysis of commercial samples

In order to demonstrate the practical usage of the biosensor, various tablets and syrup having AA were examined for estimation of AA by MIP electrode. Solution obtained by dissolution of AA tablets and syrup were subsequently diluted so that AA concentration lies in the range of calibration plot. DPVs were then recorded under exactly identical conditions. Following the proposed method, the concentration of AA in the four kinds of pharmaceutical preparations was determined. In order to validate the electrochemical detection we have compared the obtained results with both detection methods, by using the optimal chromatographic conditions. Table 2 shows the obtained data when the HPLC and DPV, using MIP electrode, are used. In this table we can observe that both detection methods were useful for analytical purposes.

### 2.9. Reproducibility of the MIP electrode

To verify the reproducibility of molecularly imprinted polypyrrole modified pencil graphite electrode, the peak current response of AA was determined with five electrodes which produced under the same conditions. The fabrication reproducibility of five biosensors shows an acceptable reproducibility, with a relative standard deviation of 4.5% for the steady-state current obtained in the AA concentration of 2.0 mM.

## 3. Experimental Section

### 3.1. Chemicals and Reagents

Dopamine (≥98.5%), L-ascorbic acid (>99.5%), uric acid (>99.5%) and D-glucose (>99.5) were obtained from Fluka (Steinheim, Germany). Potassium dihydrogen phosphate (>99.5%, Merck, Darmstat, Germany), potassium hydrogen phosphate (≥98.5%, Sigma-Aldrich, Steinheim, Germany), lithium perchlorate (98%, Lancaster, Morecambe, England) and other reagents commercially available as analytical grade and used without further purification. Pyrrole (98%, Aldrich, Steinheim, Germany) was distilled repeatedly under vacuum until a colorless liquid was obtained and then kept under nitrogen in the dark at 4 ºC. Stock AA and buffer solutions were prepared by using ultra-pure deionized water (Sartorius). Bevitin C (Abfar Ilac San.), Aspirin+Vitamin C (Upsa Conseil), Supravit (Roche) and C-PLAN (Polifarma) were purchased from a local pharmacy. Freshly prepared solutions of AA were prepared each day owing to its low stability.

### 3.2. Apparatus

Electrochemical studies were performed using an Autolab PGSTAT 100 potentiostat-galvanostat controlled by a GPES 4.9 software package (Ecochemie, The Netherlands). A three electrode system was used for all measurements; a pencil graphite electrode as the working electrode and a Pt auxiliary electrode. All measurements carried out with an Ag/AgCl reference electrode. Chromatographic measurements were carried out using a Dionex DX100 ion chromatograph with 25 µl sample loop and an anion-separation column (Dionex IonPac AS11-HC). The column is a 9−µm macroporous resin bead, consisting of ethylvinylbenzene cross-linked with 55% divinylbenzene. The anion exchange layer is functionalized with a quarternery ammonium groups. The eluent used was a buffer solution of 1.0 mM NaOH The eluents were filtered through a 0.45 µm syringe type filter (Millipore) and then degassed for 15 min. The optimum flow-rate of the eluent was 1 mLmin^−1^. An IONcheck45 model (Radiometer, France) pH-Ion meter was employed for pH measurements.

### 3.3. Preparation of MIP and NIP electrodes

A Noki pencil model 2000 (Japan) was used as a holder for graphite leads (Tombo, HB, 0.5 mm diameter, Japan). Electrical contact with the lead was obtained by soldering a metallic wire to the metallic part. PGEs were washed with water and acetonitrile to remove the impurity and dried at room temperature before the experiments. Then, PGE was immersed the polymerization solution. The MIP was obtained by electrodeposition on the surface of the PGE using cyclic voltammetry in the potential range between −0.60 and +0.80 V during seven cycles (scan rate: 100 mV/s) in aqueous solution of 0.1 M LiClO4, 0.025 M pyrrole and 0.020 M AA. After the electropolymerization process, the embedded AA were then extracted to give a surface complimentary in shape and functionality to the original template AA. The extraction was carried out by immersing the MIP film-coated electrode into 0.05 M phosphate buffer solution with agitation provided by a magnetic stirrer. A control electrode (non-imprinted polymer modified electrode, NIP) was prepared in every case under the same experimental condutions but without adding the AA, to check the reliability of the measurements.

### 3.4. Electroanalytical measurements

Differantial pulse voltammetric measurements were carried out in a three electrode cell, in 0.1 M KCl + 0.05 M phosphate buffer at pH 8.5. Before the measurements, electrolytic solutions were purged with nitrogen for 5 min. Current measurements were performed using differential pulse voltammetry (DPV) in the potential range between -0.30 and 0.40 V. To record differential pulse voltammograms, the following instrumental parameters were used: step potential 8 mV, modulation amplitude 50 mV; scan rate 15 mV/s. All electroanalytical measurement were made at room temperature.

### 3.5. Sample preparation

Various commercial pharmaceutical tablets and syrup having AA were examined for estimation of AA. The tablets were ground to a powder and then dissolved in water. The solution was sonicated for 10 min and filtered. An aliquot of appropriate volume of stock solution was transferred into 250 mL volumetric flask. The syrup was transferred to a 100 mL flask. All the samples were further diluted with buffer solution (pH: 8.5) in order that the concentration of AA was in the working range. The samples were then spiked with appropriate amount of AA for experiments.

### 3.6. The structure of interferents

**Scheme 1.**
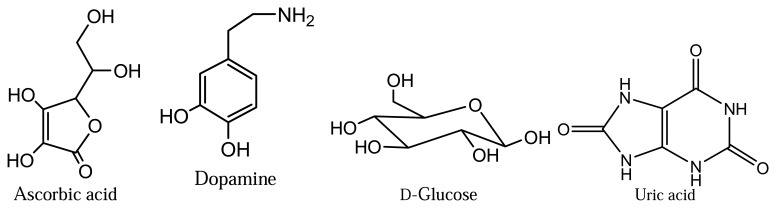
The structure of ascorbic acid, dopamine, D-glucose and uric acid.

To investigate the selectivity of the MIP and NIP electrode analyses were carried out in the presence of different interfering molecules. The structure of ascorbic acid and some possible interfering substances such as dopamine, D-glucose and uric acid are given in Scheme 1.

## 4. Conclusions

The molecularly imprinted polypyrrole modified pencil graphite electrode has been successfully applied as a sensor for fast and accurate determination of ascorbic acid in standards and some pharmaceutical samples. The advantages of very simple instrumentation and easy preparation of the proposed sensor make the system useful in constructing simple devices for determination of ascorbic acid. Now, our laboratory is processing the development of novel selective potentiometric polypyrrole detection system for AA measurements.

## Figures and Tables

**Table 1.** Effect of interferents on the DPV response of 0.50 mM ascorbic acid at the MIP electrode.

**Interferent**	**Concentration of interferents (mM)**	**Change in current response (µA)**^a^

Glucose	0.5	0.80
	2.5	5.56
	5.0	0.80

Dopamine	0.5	−4.31
	1.0	−8.68
	2.5	−10.4

Uric acid	0.5	−0.65
	1.0	−2.55
	2.5	−5.30

a The current in the absence of any interferent was 50 µA

**Table 2.** Determination of AA in pharmaceutical preparations using molecularly imprinted polypyrrole modified pencil graphite electrode.

**Tablet/syrup name** *(Company name)*	**Stated** **content**	**Detected** **content**^a^	**% R.S.D.**^a^ **(n=3)**	**Detected** **content**^b^	**%R.S.D.**^b^ **(n=3)**

**Bevitin C** *(Abfar İlaç San.)*	0.500^c^	0.481^c^	1.26	0.496^c^	0.85
**Aspirin+VitaminC** *(Upsa Conseil)*	0.500^c^	0.477^c^	0.67	0.245^c^	0.44
**Supravit** *(Roche)*	45.1^d^	43.8^d^	0.81	45.9^d^	0.61
**C-Plan** *(Polifarma)*	0.500^c^	0.465^c^	1.62	0.494^c^	0.90

a by DPV method

b by IC method

c g/tablet

d mg/100 mL
